# Motion Analysis of the Wrist and Finger Joints in Sport Climbing

**DOI:** 10.3390/bioengineering11040370

**Published:** 2024-04-12

**Authors:** Gabriella Fischer, Micha Schneeberger, Stefan Andreas Petter, Anne-Gita Scheibler, Peter Wolf, Maurizio Calcagni, Andreas Schweizer, Lisa Reissner

**Affiliations:** 1 Institute for Biomechanics, Department of Health Sciences and Technology, ETH Zurich, 8092 Zurich, Switzerland; 2 Division of Plastic Surgery and Hand Surgery, University Hospital Zurich, 8091 Zurich, Switzerland; maurizio.calcagni@usz.ch; 3 Department of Orthopedics, Balgrist University Hospital, 8008 Zurich, Switzerlandandreas.schweizer@balgrist.ch (A.S.); lisa.reissner@balgrist.ch (L.R.); 4 Sensory-Motor Systems Lab., Department of Health Sciences and Technology, ETH Zurich, 8092 Zurich, Switzerland; peter.wolf@hest.ethz.ch

**Keywords:** climbing, kinematics, 3D analysis, hand biomechanics, injury & prevention, kinetics

## Abstract

Climbing is a fast-growing sport, with one of the most common injuries being a rupture of the finger flexor tendon pulley. The strain on pulleys increases as finger joints flex. However, to our knowledge, no study has conducted a kinematic analysis of climbers’ fingers. Thus, this study aimed to examine finger kinematics during typical climbing tasks. Eleven elite climbers performed a sequence of four climbing moves, which were recorded by an optical motion capture system. Participants used crimp, half-crimp, and open-hand grips for three trials each, with the fourth condition involving campusing using any grip except crimp. Mean proximal interphalangeal joint (PIP) flexion during the holding phase was 87° (SD 12°), 70° (14°) and 39° (27°) for the crimp, half-crimp and open-hand grip, respectively. Hence, inter-individual PIP flexion ranges overlap between different gripping conditions. Two different movement patterns emerged in the open-hand grip, possibly influenced by the use of the little finger, leading to varying degrees of flexion in the middle and ring fingers. Avoiding little finger usage in the open-hand grip may reduce load during pulley rupture rehabilitation. The implications of PIP joint angle variability on individual pulley injury risk or prevention warrant further investigation. Motion capture proved effective for understanding finger kinematics during climbing and could guide future studies on pulley injury risk factors.

## 1. Introduction

Although sport climbing had its first appearance at the Olympic Games in Tokyo in 2021, only little research has been done in the field of motion analysis in sport climbing so far. Previous studies investigated the movement of the body center of mass [1,2,3], full body movement patterns and climbing behavior [4,5,6,7], joint angle changes of the elbow and knee during climbing [3], and the influence of different foot positions on the positions of other joints [8]. These studies focused on describing and comparing movement characteristics of beginners and advanced climbers. We are not aware of any studies focusing specifically on the motion of finger joints and wrist joints during sport climbing. Such a focus might be of importance, though, as finger injuries in general and flexor tendon pulley ruptures in particular account for the majority of all injuries in this increasingly popular sport [9,10,11]. However, since 3D motion analysis of small joints in general is challenging, and it has not yet been previously applied to examine the finger joints while climbing, the feasibility needs to be confirmed. In this context, sufficient marker visibility and repeatable motion patterns are a prerequisite for meaningful data interpretation.

The main function of the pulley system is to guide the flexor tendons along the phalanges. The A2 (overlying the proximal aspect of the proximal phalanx) and the A4 (overlying the mid-portion of the middle phalanx) are most important in preventing bowstringing of the flexor tendons and are most affected by pulley ruptures. Different factors contributing to pulley injury have been investigated. In general, the strain on the pulley is dependent on finger joint angles, with more flexion of the finger joints leading to a larger deflection of the flexor tendons and, therefore, more strain on the pulley. In climbing, pulley ruptures have been associated with the so-called crimp grip, where the proximal interphalangeal (PIP) joints are flexed about 90° while the distal interphalangeal (DIP) joints are hyperextended [11,12]. In this finger position, the large tendon deflection at the A2 and A4 pulley edges leads to a maximal load on the pulley [13]. In contrast, the half-crimp or open-hand grip positions are defined by less flexed PIP joints, which are associated with smaller strain in the pulley. However, the finger kinematics when climbing in these different grip positions have not been quantified so far, and therefore, it is not known to what extent the joint angles differ in different grip types and between different climbers.

Joint angles and their kinematics thus play a role in the mechanism of finger injuries during sport climbing. Nevertheless, the current knowledge on risk factors contributing to pulley overload is mainly based on questionnaires (e.g., about the incident of injury, climbing habits) [9,10], clinical examinations [9,14,15] or biomechanical studies on cadaveric hands [16,17,18]. Analyzing finger joint and wrist motion during sport climbing could contribute to a better understanding of the injury mechanisms from a biomechanical point of view.

The aim of this study was to find out whether optoelectronic motion capture is a practicable method for the motion analysis of wrist and finger joints in the context of sports climbing. Further, we wanted to accurately describe the joint positions and flexion-extension motions for the grip positions most commonly used during sport climbing.

## 2. Materials and Methods

### 2.1. Participants

Finger joint and wrist motions of 12 healthy sport climbers at a local climbing gym were assessed by means of an optoelectronic motion capture system while performing climbing moves. As we were interested in the finger movements of experienced climbers, we have set a self-reported redpoint climbing level (climbing a route or boulder without rest after rehearsal) of at least French 8a for lead climbing or Fontainebleau (Fb) 7B+ for bouldering. As different climbing grading scales exist, the International Rock Climbing Research Association (IRCRA) proposed a method for reporting climber characteristics to improve consistency and facilitate comparison [19]. We have converted the self-reported climbing levels in accordance with the IRCRA reporting scale (Table 1). According to the IRCRA classification, one was a higher elite (≥28), eight were elite (24–27.5), and two were advanced-level (18–23.5) climbers, whereby only climbers with an IRCRA score ≥ 23 could participate in this study.

Exclusion criteria included any inflammatory joint diseases or other conditions that could have impaired normal climbing performance. Data from one climber had to be excluded from the analysis due to technical problems during kinetic data acquisition. Of the remaining 11 climbers (10 men, 1 woman) (see Table 1), the mean (standard deviation) BMI was 22.0 kg/m^2^ (1.3 kg/m^2^), and the mean highest self-reported redpoint level (higher value in lead climbing or bouldering) on the IRCRA scale was 26.2 (1.6).

All participants provided written informed consent for their data to be used for this analysis. According to the local ethics committee, no approval of this study was necessary (BASEC-Nr. 2018-01128). All methods were performed in accordance with the Declaration of Helsinki.

### 2.2. Setup

All measurements were carried out in the motion analysis laboratory of the Department of Plastic Surgery and Hand Surgery of the University Hospital of Zurich, Switzerland.

#### 2.2.1. Climbing Wall

**Figure 1:**
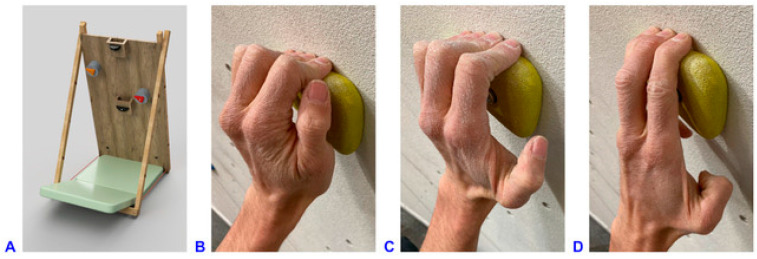
(**A**) 3D representation of the climbing wall used in the experiment. Climbing wall dimensions: 100 cm × 200 cm, −20° inclination angle, removable square board of 86 cm × 74 cm at the central bottom of the wall. Dimensions and position coordinates of the holds (@ refers to position of the holds with respect to the coordinate system origin at the lower left corner of the climbing wall): left foothold: 20 mm deep @15 cm/25 cm; right foothold: 20 mm deep @85 cm/25 cm; starting hold: 180 mm wide, 36 mm deep @50 cm/110 cm, placed on wooden box 105 mm deep; right instrumented hold (red): 110 mm wide, 23 mm deep, 25° incut angle, @75 cm/130 cm, placed on load cell unit 105 mm deep; left instrumented hold (orange): 110 mm wide, 23 mm deep, 25° incut angle, 15/160 cm, placed on load cell unit 105 mm deep; top hold: 140 mm wide, 75 mm deep @50 cm/198 cm. (**B**) Crimp grip with flexed PIP (approximately 90°) and hyperextended DIP joints, without using the thumb. (**C**) Half-crimp grip with only slightly flexed or even straight little finger, leading to less PIP joint flexion and not fully hyperextended DIP joints. (**D**) Open-hand grip with straight index finger, with (four-finger open-hand) or without (three-finger drag, shown in picture) little finger on the hold.

A custom-made freestanding overhanging climbing wall was equipped with four handholds and two footholds (Figure 1). An inclination angle of 20° was selected to provide adequate difficulty of the task for experienced climbers. The starting hold was a ledge, and the top hold was a fairly big jug. The two instrumented holds in between were identical edges.

#### 2.2.2. Data Acquisition: Interaction Force Measurement System and Motion Capture System

To assess finger joint and wrist motion specifically during loaded conditions, the two intermediate holds were instrumented with self-developed load cells recording forces and moments in all spatial directions with a sampling rate of 1000 Hz. The instrumentation has already been described elsewhere [20].

Kinematic data of finger and wrist motion were collected using an optoelectronic motion capture system. Eleven wall-mounted infrared cameras (VICON^®^ MX3+ and VICON^®^ MX3 motion capture system, Oxford Metrics Ltd., Oxford, UK) and the corresponding software VICON^®^-Nexus (version 2.3) were used for data collection. The cameras had a resolution of 659 × 493 pixels, and recordings were carried out with a frequency of 100 Hz.

**Figure 2:**
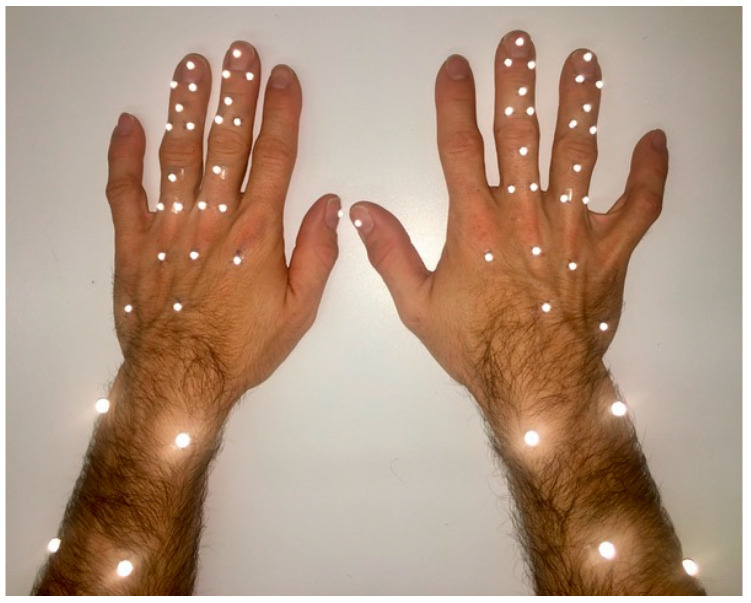
Applied marker set with 23 hemispherical 3 mm markers on the hand and 4 spherical 5 mm-markers on the forearm of each hand. The kinematic evaluation was based on 8 segments per hand defined by the marker clusters on the forearm, palm, and the proximal, intermediate and distal phalanges of the fingers. The flexion axis of the joints was determined by a functional approach.

As a high number of markers in a small volume poses a challenge for simultaneous motion tracking [21] and as pulley injuries occur almost exclusively on the middle or ring finger [9,12,14,22], we evaluated only these two fingers. Three reflective skin markers (hemispherical, 3 mm in diameter) were triangularly arranged on each phalanx of the middle and ring finger (Figure 2). The same marker size was used for the heads of the metacarpal bones II, III and IV and the bases of the metacarpal bones III and V on the back of the hands. Bigger markers (spherical, 5 mm in diameter) were placed on the styloid processes of radius and ulna, as well as on the bodies of radius and ulna a quarter and a third of forearm length further proximally, respectively.

### 2.3. Experimental Protocol

#### 2.3.1. Basic Motion Tasks

In order to calculate the functional joint centers and axes of each joint, the following basic motion tasks were recorded as previously described [23]: static neutral reference position; wrist flexion–extension; wrist radial–ulnar deviation; adduction–abduction of all fingers in the metacarpophalangeal (MCP) joints; adduction–abduction of the middle finger separately in the MCP joint; full finger flexion–extension; distal finger flexion–extension (only PIP and DIP joints). For a detailed description and illustration of the movements, we refer to the previous publication [23]; further kinematic evaluation is described in Section 2.4.2. Four trials per task were recorded to calculate the mean functional joint centers and axes of each joint.

#### 2.3.2. Climbing Tasks

The climbing tasks that were generally requested consisted of a sequence of four climbing moves. Starting with both hands on the starting hold, the participants had to move to the right instrumented hold with the right hand, continue to the left instrumented hold with the left hand, reach the top hold with the right hand, and match the top hold with the left hand (Figure 1A).

The two instrumented holds had to be grasped in three different finger positions, i.e., by the crimp, half-crimp, and open-hand grip (Figure 1B–D) while standing on the two footholds. The fourth test condition requested campusing up the handholds without using the footholds. The square board at the bottom of the wall supporting the two footholds was removed for this task (Figure 1A), and the participants were allowed to use any but the crimp grip (due to the increased risk of injury). All handholds were horizontally aligned. The participants wore climbing shoes. Three to four successful trials for each task were recorded (first half-crimp, then crimp, open-hand and campusing task). Criteria for a successful trial were as follows: the sequence of the four climbing moves completed, the climber subjectively confirmed the required grip task, no marker dropped, recording of the entire trial with both systems and no use of foot contact in the campusing task. The motion capture and the interaction force measurement systems were synchronized for every trial using a trigger.

### 2.4. Data Processing and Analysis

#### 2.4.1. Kinetic Data

Analysis of the kinetic data was performed in MATLAB^®^ R2019b (The MathWorks Inc., Natick, MA, USA). The three force components measured by the instrumented holds were summed up into a resulting force. Each kinetic data set was reduced using the downsample MATLAB function to account for the different sampling rates of the two measurement systems. The kinetic curves were smoothed with the moving average filter (window size 4). The measured force was normalized to the body weight (BW) of each climber.

#### 2.4.2. Kinematic Data

VICON-Nexus software (2.9.2) was used for 3D position reconstruction and labeling of the markers, including interpolation of small gaps. The obtained data were imported into MATLAB software for further analysis. Segmental kinematic evaluation was based on marker clusters and rigid body assumption, similar to previous studies of our research group [23]. For the current kinematic hand model, we defined eight segments per side and adapted a functional approach from List et al. [24] to calculate the joint centers and axes of the wrist and finger joints [23]. In contrast to our previous protocol, which used a reduced marker concept for the fingers [23], in the current study, a full 3D approach was applied, including functional determination of the flexion axes of the finger joints. Hence, the joint coordinate systems were defined based on a functional approach instead of anatomical landmarks, but their sequence in the decomposition of the relative rotation matrix was compliant with the recommendations of the International Society of Biomechanics [25], namely:

e1: Functional flexion–extension axis, fixed to the proximal segment;

e3: Fixed to the distal segment and coincident with the longitudinal axis of the distal segment (pronation–supination);

e2: Common axis perpendicular to e1 and e3 (radial–ulnar deviation).

Joint kinematics were calculated according to Grood et al. [26]. In this study, we focussed on flexion–extension joint motion (unless otherwise noted); however, radial–ulnar deviation and longitudinal rotations were also considered for the description of the joint position during the holding phase. The resultingg joint angles were checked again for errors by detecting sharp positive or negative peaks in the angle curves. Differences of ≥7° between the two frames were considered to require further checking. In the first step, the corresponding trials were checked for labeling and fitting errors and corrected back in the VICON acquisition data using a rigid body interpolation built into the VICON-Nexus software if the source of error could be identified. Afterward, joint angle calculation was repeated for the affected trials. Partial occlusion of markers can lead to noise in the detected marker trajectory, leading to sharp positive or negative peaks in the corresponding angle curves. In a second step, the remaining outlier peaks were detected using the moving median method (window size 5, difference of >2 scaled mean absolute deviations to the local median) and cut out. The resulting gaps were interpolated with the “filloutliers” MATLAB function using the piecewise cubic hermite interpolating polynomial (pchip) method. Missing values of up to 20 frames due to marker occlusion were interpolated with the ”fillmissing” MATLAB function using the pchip method.

#### 2.4.3. Time Normalization of Kinetic and Kinematic Data

**Figure 3:**
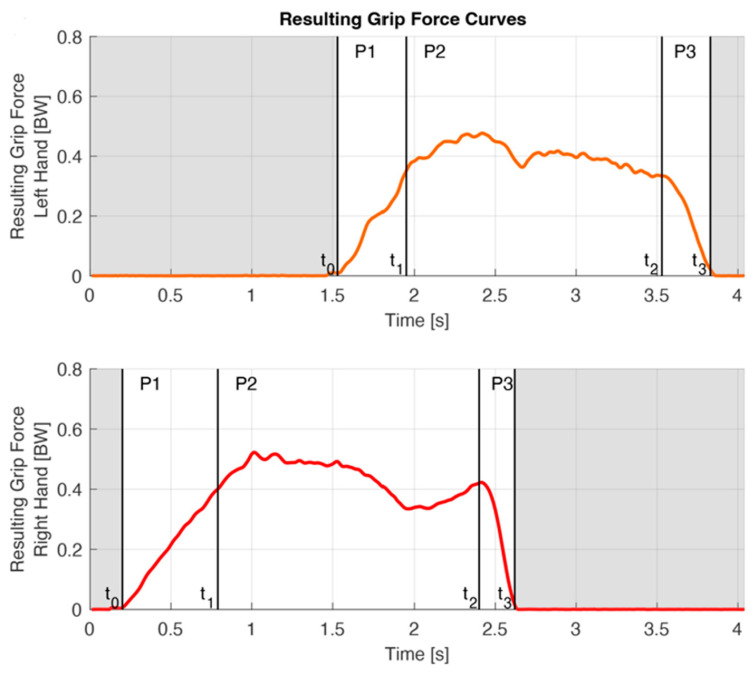
On the basis of the force curves, the handhold contact period was divided into three phases: the grabbing, holding and releasing phase (P_1_–P_3_) bordered by four phase transitions (t_0_–t_3_), here shown for a crimp grip trial. The red line refers to the resulting grip force of the right hand, the orange line to that of the right hand. Initial contact t_0_ was defined as the penultimate frame before 15N was exceeded for the first time during a trial or, in the case of a regrip, as the point of time of the local minimum of the force curve. Left t_1_ was determined by the moment of the local load minimum on the right hand between left t_0_ and right t_2_. Right t_1_ was assumed based on the average duration of P1 of the left hand, as the starting hold was not instrumented. (Note that right and left P_1_ durations are different in a single trial, as the right P_1_ duration was assumed based on the mean P_1_ duration of all trials of the left.) t_2_ was defined as the last moment before the slope of the kinetic curve drops below −1.5 N per frame (0.01 s). t_3_ was defined as the data point two frames after the interaction force fell below 15 N for the last time during a trial.

As climbers remained in a certain position for varying periods of time, time normalization was needed in order to allow comparison of trials. We subdivided the handhold contact period into three phases delimited by four phase transitions (Figure 3).

The grabbing phase (P1) begins with the initial contact on the handhold (t0). The load increases quickly while grabbing the hold and shifting the center of mass to this side. P1 ends when full control of the handhold is established (t1).

- t0 was defined as the penultimate frame before 15N was exceeded for the first time during a trial. If readjustment of the fingers was necessary due to suboptimal finger placement on the handhold, a temporary load release was seen on the force curve. All trials were checked for such regrips, according to Bauer et al. [20]. In the case of a regrip, the point of time of the local minimum of the force curve replaced t0.
- t1 of the lead hand can be assumed when the load on the contralateral hand is maximally reduced. Hence, left t1 was defined as the moment of the local load minimum on the right hand between left t0 and right t2 (Figure 3). As the starting hold was not instrumented, right t1 could not be determined likewise. Instead, we had to assume that the duration of the right P1 was the same as the mean P1 duration of all trials of the left hand, which, in our view, was a reasonable assumption as the moves were comparable.

During the holding phase (P2), the contralateral hand reaches for the next handhold and establishes handhold control there. The center of mass is then shifted back to the contralateral side.

- t2 was defined as the last moment, where the slope of the kinetic curve was bigger than −1.5 N per frame (0.01s).

The releasing phase (P3) starts with the onset of a rapid load drop (t2) and ends with the handhold clearance (t3).

- t3 was defined as the data point two frames after the interaction force fell below 15N for the last time during a trial.

For each trial, kinetic and kinematic data arrays were time normalized for each phase separately.

#### 2.4.4. Metrics of Interest

Marker visibility was assessed by a graphical representation of the movement sequence for each individual climber. For this consideration, a trial was considered valid if marker visibility was present for at least 75% of the contact phase to calculate the joint angle.

The intra-subject SD of the joint angle over the whole contact time, as well as at the phase transitions t1 and t2, served as a measure of variability within a participant. Variability was calculated for all trials in one condition of a participant and then averaged over all climbers.

The range of motion (ROM) was calculated in each phase for the wrist and the MCP, PIP and DIP joints of the ring and middle finger in flexion–extension, radial–ulnar deviation and longitudinal rotation.

To describe the grip position during the holding phase, the mean P2 joint angle was calculated for every trial, with less than 25% of the data missing during P2. The mean over all trials was calculated for each individual climber and then averaged over all participants. Joint angles were provided for all three anatomical planes.

Furthermore, the number of participants for whom kinematic parameters could be extracted was reported and given for each joint and climbing task.

#### 2.4.5. Statistics

Microsoft^®^ Excel for Mac version 16.61.1 (Microsoft Corporation, Redmond, WA, USA) and MATLAB^®^ R2019b (The MathWorks Inc., Natick, MA, USA) were used for statistical analysis. Unless otherwise specified, data are reported as mean (SD). As the finger position might depend on the length ratios of the fingers, the correlation between mean ring finger PIP joint angle during the holding phase and little to ring finger length ratio was checked using Spearman’s rank correlation coefficient (r) after visually verifying that the data was not normally distributed using a quantile-quantile plot. The alpha value was set at *p* < 0.05. The mean measured joint angles during the holding phase were compared between the two hands for each measured joint and task and between the corresponding joints of the middle and ring finger, using a paired two-tailed *t*-test or a Wilcoxon rank sum test. Normality and equal variance of the data were checked before performing the statistical analysis using a Shapiro–Wilk parametric hypothesis test and Bartlett’s test, respectively. Only participants with valid data for both corresponding variables were included in the statistical comparisons. Marker visibility of at least three markers per segment is required to calculate the joint angles. The mean angle during the holding phase was considered valid, if the marker visibility allowed to calculate the corresponding joint angle during at least 75% of the holding phase. Statistical tests were only performed if data of at least five participants was available.

## 3. Results

### 3.1. Data Quality and Feasibility Criteria for Motion Analysis in Climbing

All eleven participants performed the crimp, half-crimp and open-hand grip tasks. One climber (CLIM 0002) was not able to perform the campusing task. The joint angles of the left ring finger MCP and PIP of CLIM 0010 and of the left middle finger PIP and DIP of CLIM 0004 could not be calculated due to missing markers in the reference trial. Marker visibility was best during the open-hand grip, followed by the half-crimp and the crimp grip, and was worst during the campusing task (Figure A3, Figure A4, Figure A5 and Figure A6, Table 2). The more proximal the joint of interest, the better its marker visibility. Further, marker visibility was best during P2 and worst during P3 (Figure A3, Figure A4, Figure A5 and Figure A6, Table 2).

The intra-subject repeatability of the climbing motion between different trials, represented by the pooled intra-subject SD over the entire contact time, ranged between 1.5° and 7.2° for both fingers and all joints. The crimp task was the condition with the highest intra-subject repeatability (2.5°, SD 0.5°), whereas the campusing task was executed with more intra-subject variability (4.1°, SD 1.4°). A total of 10.7% of the individual intra-subject SDs were >5°, only 3.5% > 7°. In terms of the angular values at the time points of the phase transitions (t1, t2), the pooled intra-subject SD was 3.2° and 2.9°, respectively.

### 3.2. Graphic Representation of the Movement Sequences

In almost all joints and conditions, at least three participants were evaluated, only in the crimp condition and only for DIP right, where not enough data was available due to marker occlusions.

**Figure 4:**
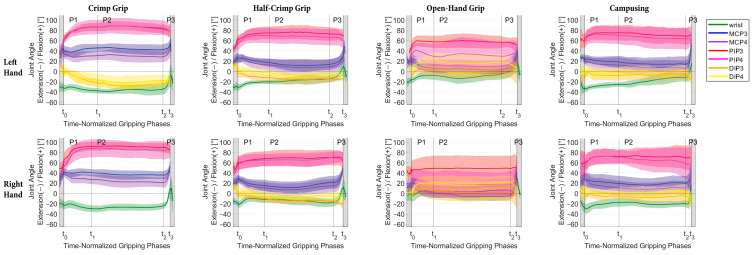
Mean and SD of flexion–extension angle progress over all participants during the time-normalized gripping phases (P_1_ = gripping, P_2_ = holding, P_3_ = releasing) for the joints of the left (**top** row) and right hand (**bottom** row) for the different grip positions (minimum n = 3 for mean and SD calculation).

During the grabbing phase (P1), PIP joint flexion and MCP joint extension occurred in most tasks, as represented by an average PIP and MCP joint angle change of 18.1° and −9.3°, respectively (Figure 4). When crimping, the wrist made an extending movement (−5.2°), whereas in the other tasks wrist flexion could be observed (8.3°). During the releasing phase (P3), averaged over all conditions, wrist and MCP joint flexion of 26.2° and 25.0°, respectively, and 5.6° PIP flexion occurred (Figure 4).

Averaged over all participants, tasks and joints, the flexion–extension ROMs during the grasp, holding and releasing phases were 18.6° (3.6°), 9.5° (1.5°) and 15.2° (9.9°), respectively. The largest movements (ROM > 20°) were found for the PIP3 and PIP4 during the grasping and for the wrist, MCP3 and MCP4 during the releasing phase (Table 2).

**Figure A1:**
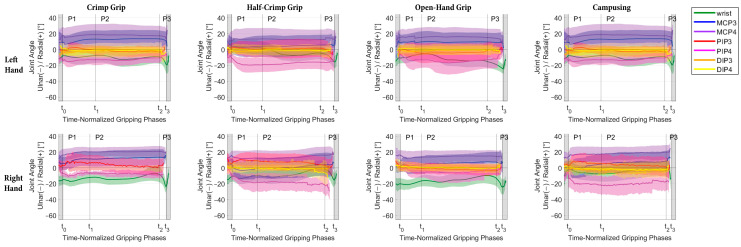
Mean and SD of radial–ulnar deviation angle progress over all participants during the time-normalized gripping phases (P_1_ = gripping, P_2_ = holding, P_3_ = releasing) for the joints of the left (**top** row) and right hand (**bottom** row) for the different grip positions.

**Figure A2:**
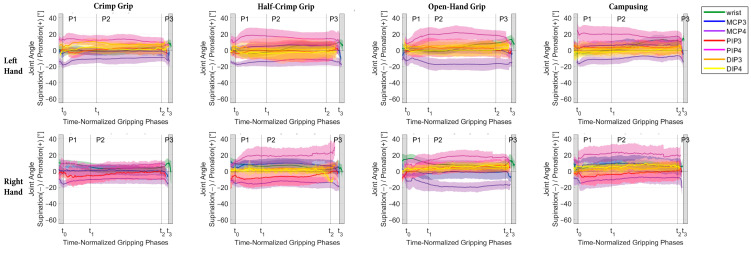
Mean and SD of pronation–supination angle progress over all participants during the time-normalized gripping phases (P_1_ = gripping, P_2_ = holding, P_3_ = releasing) for the joints of the left (**top** row) and right hand (**bottom** row) for the different grip positions.

The radial–ulnar deviation ROM was 8.4° (2.3°), 6.9° (2.5°) and 5.5° (3.6°) for P1, P2 and P3 (Figure A1, Table 3 and Table A1), respectively and the corresponding values for the pronation–supination were 10.8° (2.0), 8.9° (0.8°) and 6.7° (1.8°), respectively (Figure A2, Table 4 and Table A2).

### 3.3. Grip Positions during Holding Phase (P2)

#### 3.3.1. Finger Position Variability during Holding Phase (P2)

Regarding the finger position variability during P2 within a participant, the mean pooled within-subject SD of the P2 joint positions was 2.2° (SD: 2.0°, range 0.2°–15.7°) for the crimp grip, 2.2° (SD: 1.5°, range 0.2°–8.6°) for the half-crimp grip, 2.5° (SD: 2.7°, range 0.3°–23.4°) for the open-hand grip, and 3.4° (SD: 3.3°, range 0.3°–19.3°) for campusing. 10.1% of all within-subject SD values were above 5°, 3.8% above 7°, and 75% were <3°.

As to the overall finger position variability, the between-subjects SDs of the mean P2 joint positions range between 5.8° and 16.4° for the crimp grip, 6.0° and 15.2° for the half-crimp grip, 7.6° and 29.0° for the open-hand grip, and 6.5° and 19.9° for campusing (Table 5).

#### 3.3.2. Description of Flexion–Extension Joint Angle Positions

During the crimp grip, average PIP joint flexion was 86.5° and DIP (hyper)-extension was 20.1° (Table 5 and Figure 4). In the half-crimp grip, the positions of all joints were closer to neutral than in the crimp grip position, which is most pronounced in the MCP joints. The grip position chosen for the campusing task was most similar to the half-crimp grip. In the open-hand grip, larger SD bands (>20°) were found for the PIP joint angles (Table 5 and Figure 4).

**Figure 5:**
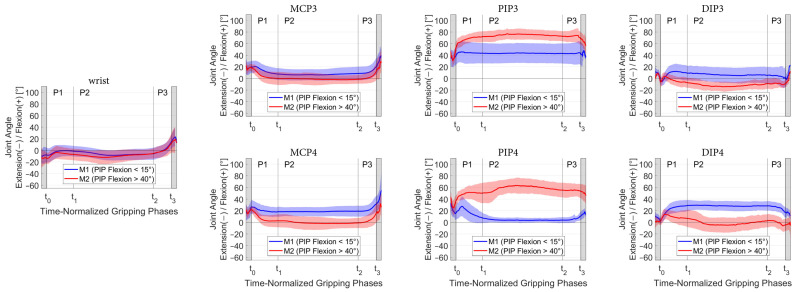
Mean and SD of the finger joint and wrist angles divided between the two methods during open-hand grip classified by PIP flexion angle, whereby trials with PIP flexion < 15° were assigned to group M1 (blue) and trials with PIP flexion > 40° to group M2 (red).

**Figure A3:**
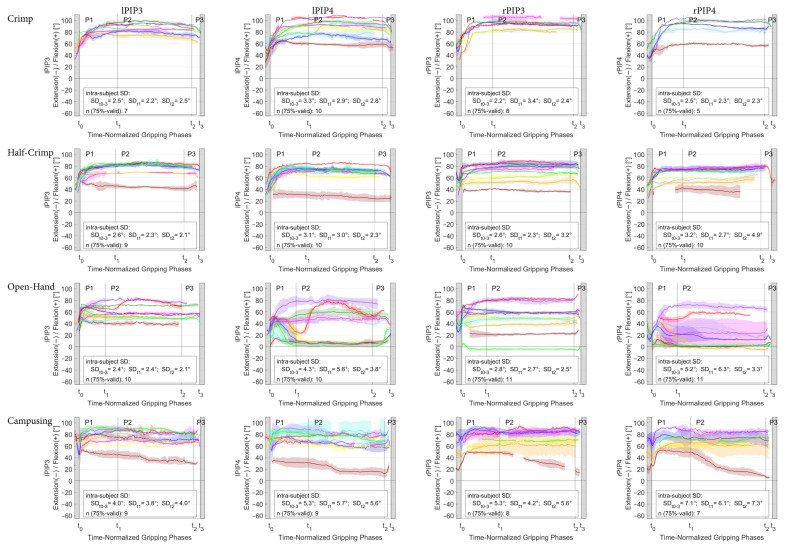
Mean and SD flexion–extension angle in the PIP joints of the middle and ring fingers plotted for each individual climber (for the legend color, see Table 1) during the time-normalized gripping phases (P_1_ = gripping, P_2_ = holding, P_3_ = releasing) for the crimp, half-crimp, open-hand grip and campusing task. The pooled intra-subject repeatability between the different trials is indicated for the whole contact phase (SD_t0–3_) as well as at the phase transitions (SD_t1_ for the beginning and SD_t2_ for the end of the holding phase). n(75%-valid) refers to the number of subjects with valid data for at least 75% of the contact phase.

Looking at the individual climbers, two different movement patterns could be identified in the open-hand grip (Figure A3 and Figure 5). Six participants (CLIM 0001, CLIM 0006-7, CLIM 0009-11) used a more extended method, where the ring finger PIP joint was slightly extended or flexed less than 15° (Figure A3, lPIP4 and rPIP4 during open-hand grip). On the other hand, four participants used a more flexed open-hand grip method with marked flexion of the ring finger PIP joint ranging from 23.4° to 78.5° (Figure A3). However, one participant (CLIM 0002) used the extended method for the right, and the flexed method for the left hand, and one participant (CLIM 0008) used the extended method in his first two attempts and the flexed method in the third attempt for the right hand whereas the flexed method was consistently observed for his left hand. Only CLIM 0004 cannot be assigned to either of the two patterns for the right hand, as this climber starts in a flexed position and changes to a more extended position later during the holding phase. No significant correlation between ring finger PIP joint angle and little to ring finger length ratio could be found (left hand: r = 0.01, *p* = 0.98; right hand: r = 0.04, *p* = 0.92). The individual DIP joint angles during the open-hand grip ranged from 18.4° extension to 39.7° flexion (Figure A6).

Averaged within participants, the mean PIP joint angles during P2 ranged from 58.3° to 106.2° for the crimp, 27.9° to 86.9° for the half-crimp, −4.3° to 82.0° for the open-hand grip and 20.0° to 91.4° for campusing (Table 6).

#### 3.3.3. Radial–Ulnar Deviation and Axial Rotations

During all grip modalities, the wrist and PIP4 were slightly (<20°) ulnar, and the MCP joints slightly radially deviated (Figure A1). In the PIP3 and DIP joints, depending on the grip position, either a radial or ulnar deviated position was detected, likewise, the lateral deflection was smaller than in the previously mentioned joints (<7°). Statistically significant differences between the middle and ring finger were found in both hands for the radial–ulnar position of the PIP during crimp, half-crimp and campusing, as well as for the MCPs of the right hand during crimp and half-crimp. Lateral movement was similar for both hands (no statistically significant differences between the left and right side for radial–ulnar position of all joints and gripping conditions).

During all tasks, there was a slight (<8°) internal rotation within the wrist. Hence, the palm segment was slightly pronated with respect to the distal forearm marker cluster (pronation–supination of the forearm was not measured). In the open-hand grip, the left wrist was significantly less pronated than the right wrist (*p* = 0.04). Regarding axial finger rotations, supination of the MCP4 and pronation of the PIP4 were observed during all tasks. These PIP and MCP axial rotations were significantly larger in the ring finger compared to the middle finger (*p* = 0.017—*p* < 0.0001).

## 4. Discussion

### 4.1. Applicability of Motion Capture of the Hand during Climbing

To our knowledge, this is the first study to investigate finger kinematics during climbing. A key factor for the applicability of motion capture is the visibility of the skin markers. Overall, >85% of the participants had trials with good marker visibility during at least 75% of the contact time. Still, despite careful selection of the measurement setup, marker visibility proved to be challenging, especially on the distal parts of the fingers and during the releasing phase. In the crimp and the half-crimp grip, markers hidden behind the flexed PIP joints lead to a lot of missing data for the DIP joints. Data interpolation and smoothing were used to increase the amount of evaluable data. However, data labeling was time-consuming, and angle curves with larger gaps had to be excluded from further evaluation. Nevertheless, for the holding phase, which is the focus of our evaluation, we were able to extract valid trials for 100% of the participants for the wrist and the MCP3, 95% for the MCP4, 90% for the PIP3, PIP4 and DIP4 and 84% for the DIP3 (Table 5). Only in the left hand during crimp marker visibility was poor, leading to missing data for 36–64% of the participants. For all other joints and tasks, >80% of the participants had valid data during the holding phase, with an average of 93% of evaluable participants, which we consider to be good data quality. In future studies, the data quality could probably be improved by increasing the number of cameras and by using more recent cameras with higher resolution. Whether sufficient marker visibility can be achieved, however, must be considered in the context of the research question and the joint of interest.

**Figure A4:**
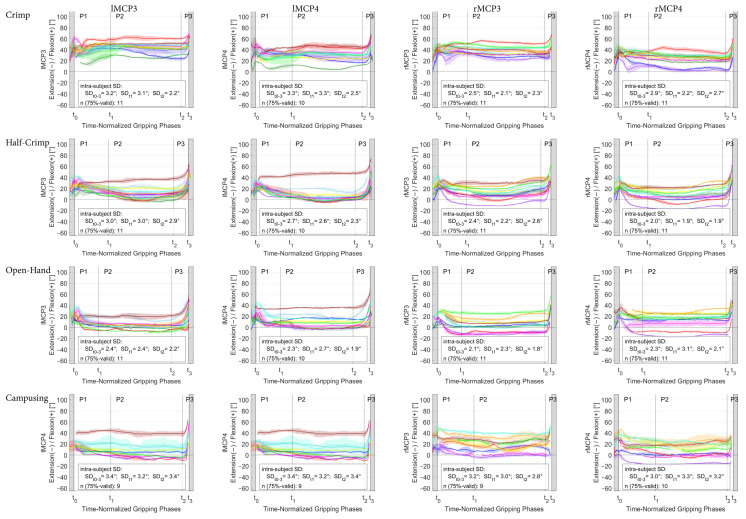
Mean and SD flexion–extension angle in the MCP joints of the middle and ring fingers plotted for each individual climber (for the legend color, see Table 1) during the time-normalized gripping phases (P1 = grip-ping, P2 = holding, P3 = releasing) for the crimp, half-crimp, open-hand grip and campusing task. The pooled intra-subject repeatability between the different trials is indicated for the whole contact phase (SD_t0–3_) as well as at the phase transitions (SD_t1_ for the beginning and SD_t2_ for the end of the holding phase). n(75%-valid) refers to the number of subjects with valid data for at least 75% of the contact phase.

**Figure A5:**
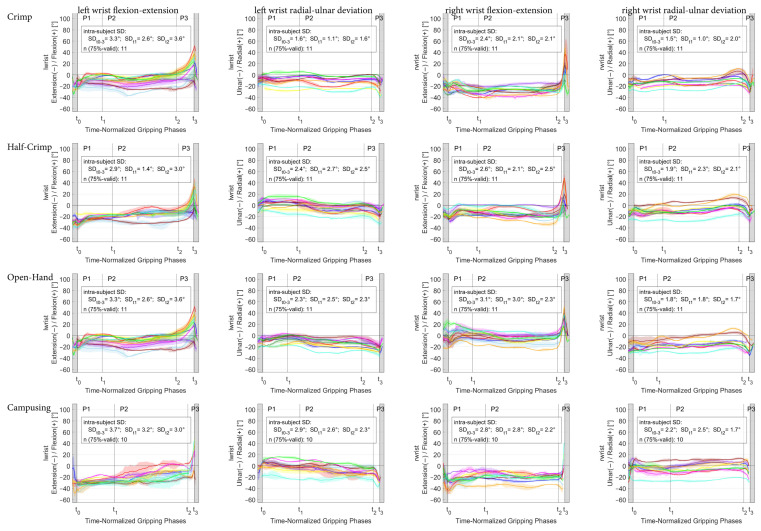
Mean and SD flexion–extension and radial–ulnar deviation angle in the left and right wrist joint plotted for each individual climber (for the legend color, see Table 1) during the time-normalized gripping phases (P_1_ = gripping, P_2_ = holding, P_3_ = releasing) for the crimp, half-crimp, open-hand grip and campusing task. The pooled intra-subject repeatability between the different trials is indicated for the whole contact phase (SD_t0–3_) as well as at the phase transitions (SD_t1_ for the beginning and SD_t2_ for the end of the holding phase). n(75%-valid) refers to the number of subjects with valid data for at least 75% of the contact phase.

**Figure A6:**
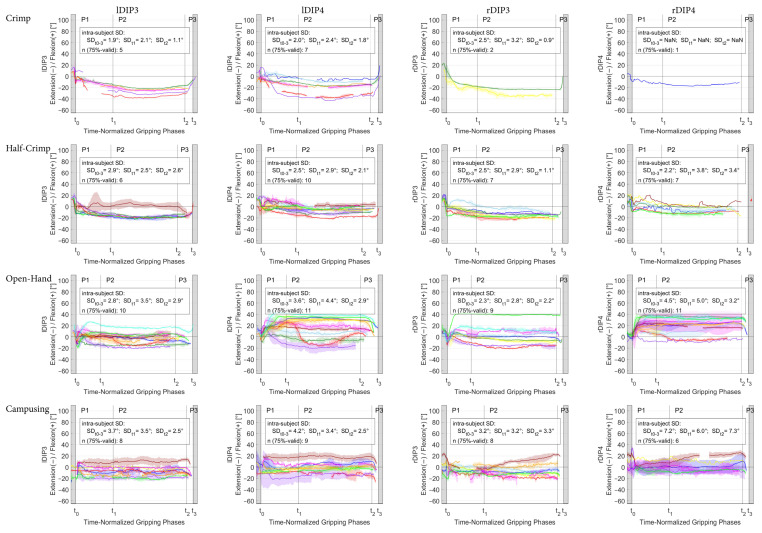
Mean and SD flexion–extension angle in the DIP joints of the middle and ring fingers plotted for each individual climber (for the legend color, see Table 1) during the time-normalized gripping phases (P1 = grip-ping, P2 = holding, P3 = releasing) for the crimp, half-crimp, open-hand grip and campusing task. The pooled intra-subject repeatability between the different trials is indicated for the whole contact phase (SD_t0–3_) as well as at the phase transitions (SD_t1_ for the beginning and SD_t2_ for the end of the holding phase). n(75%-valid) refers to the number of subjects with valid data for at least 75% of the contact phase.

Repeatable motion patterns are a prerequisite for meaningful data interpretation in motion analysis, as only a small number of trials can be evaluated to minimize the effect of fatigue due to the effort involved in data acquisition and evaluation. The joint angles between different trials of the same task varied only slightly (~3°) within a participant (Figure A4, Figure A5 and Figure A6), confirming that elite climbers demonstrate a highly repetitive pattern of finger and wrist kinematics (as long as the movement sequences are kept).

Thus, the present study shows the applicability of motion analysis in evaluating climbing movements, which could guide future studies on risk factors for pulley injuries, such as eccentric loading conditions.

### 4.2. Movement Segmentation Based on Force Measurement

Sport climbing is an acyclic activity with variable durations of the different phases of a motion sequence. In order to account for this non-cyclic character of climbing movements, the kinematic data had to be analyzed separately for the grabbing, holding and releasing phases. This could be ensured by simultaneously collecting kinetic data. The phase detection is an important factor of the data analysis because the joint angles change significantly during the grasp and release of the handle. For visual comparison of trials with different durations, normalization for time for each of these phases was performed. The high intra-subject reliability of the measured joint angles (pooled between-trial SD = 3.2° over the whole contact time and at the phase transitions t_1_ and t_2_ SD_t1_ = 3.0° and SD_t2_ = 2.6°) suggests a reasonable detection of the phases. For future kinematic studies on climbing, we also recommend subdividing the contact time into different phases; however, it does not require such an elaborate force measurement but rather a simpler instrumentation of the handles.

It was previously assumed that the holding phase is characterized by a stable hand position [27]. However, quantification of the finger and wrist movements was required to confirm a static holding phase. Flexion–extension joint angles did not vary considerably during P2 (Figure 4) but remained stable, as indicated by a mean absolute difference during P2 between 4.0° with open-hand grip and 6.2° when campusing. Hence, it was similar in all four tasks. These stable finger and wrist positions during P2 are very plausible from a biomechanical point of view. When pulling on hold for locomotion, rigid wrist and finger joints allow direct force transmission of the forces generated by the larger muscles of the upper arm, shoulder and back muscles on the wall.

The movements during P1 proved to be different from those in P3, as the grabbing hand reaches the hold from below, whereas the releasing hand leaves the hold in an upward movement. In P1, the largest movement occurred in the PIP joints, whereas in P3, movement of the hand was brought about mostly by wrist and MCP joint flexion, while not much movement occurred in the PIP joints (Figure 4 and Figure A3).

### 4.3. Grip Positions during Holding Phase (P2)

Our measurements confirm the general descriptions of the crimp [10,28,29] and the open-hand [9,28,29,30] grip flexion–extension positions in previous studies. In our detailed investigation, however, it became clear that the ranges of PIP joint flexion overlap between the different gripping conditions (Figure 4). Comparably detailed data have only been collected for the wrist so far. Thereby, it was observed that the wrist extension depended on the hold and grip position, whereby it was smallest in slope and highest in crimp [29]. A similar observation was made in our study, yet the obtained amount of extension (4°–37°) was smaller compared to 19°–57° in [29]. A possible explanation is that also the inclination of the wall (0° [29] vs. 20° in our study) and the distance between the holds/footrest influence the wrist angle. In fact, we observed a significantly higher extension in the left wrist during the crimp compared to the right, which presumably is attributed to the different body positions resulting from the asymmetric climbing task.

The grip positions were performed very similarly by the participants (intra-subject SD 2.6°), but large inter-individual differences in the applied grip positions were observed (mean inter-subject SD 12.4° over all conditions), in particular during the open-hand grip (inter-subject SD 15.4°). Remarkably, one individual even showed PIP joint angles that were similar to the crimp when advised to climb with an open-hand grip.

#### 4.3.1. Crimp Grip

The crimp grip was characterized by markedly flexed PIP joints and hyperextended DIP joints (Table 5 and Figure 4). In the crimp grip, the average of all measured PIP joint flexions of 86.7° was very close to 85.8°, where friction between the tendon and pulley has been found to be highest [31,32]. As higher friction means less holding force is required, it seems natural that climbers automatically choose PIP joint angles close to 85.8° in order to maximize the amount of friction. The finally chosen PIP joint position is a compromise, though, representing the trade-off between the highest friction possible, increase of the moment arm and potentially adoptable body position, which depends on the performed climbing move [8]. According to the literature [33], the flexion–extension moment arms at the PIP and DIP joints show a tendency to increase as they approach the flexed position. For the individual climbers, PIP joint angles ranged from 58.3°–106.2°, which in some cases was well below the 90°–100° described elsewhere [10,16,30]. In particular, one climber (CLIM_0007, Figure A2) adopted a more extended PIP position apparent in the crimp, half-crimp and campusing task.

#### 4.3.2. Open-Hand Grip

The average measured flexion angle of 39.1° (27.4°, range −4°–82°) in the PIP joints was larger and of 9.6° (17.2°, range −18°–40°) in the DIP joints was smaller than expected based on previous descriptions of the slope grip, which assumed 0–10° PIP flexion and 50–70° DIP flexion [30]. Unexpectedly, some individuals even adopted an extended position in DIP. During the open-hand grip, the PIP and DIP joint angles differed between the middle and ring finger; hence, in particular, in the middle finger, there is a considerable deviation as compared to previous descriptions of this grip position. The relatively high overall finger position variability in the open-hand grip is explained by two methods used by the participants, which differ most strikingly in ring finger PIP joint flexion (Figure 5). When adopting the grip position (P1), in climbers assigned to M1, the initial PIP flexion is followed by a significant PIP extension, in contrast to all other conditions (Figure 4 and Figure A3) where there was basically a flexion movement in the PIP joint toward the requested grip position. The position thus achieved, M1 corresponded rather than M2 to the description of the open-hand grip according to the literature [30]. Still, also in M1, the DIP4 angle of 30° was smaller (vs. 50–70° [30]), which again might be explained by different inclinations of the wall.

Although not tracked by the motion capture system, it can strongly be assumed that the difference (M1 vs. M2) was a result of whether the little finger was used (four-finger open-hand) or not (three-finger drag). The missing correlation between ring finger PIP joint angle and little to ring finger length ratio indicates that these methods represent a personal habit rather than being determined by relative little finger length. In our clinic, patients are advised to restart climbing after pulley injuries using only the open-hand grip in order to keep the load on the convalescent pulley as low as possible. Careful instruction to use the three-finger drag and not the four-finger open-hand grip is probably beneficial for optimal rehabilitation. As the bowstringing of the flexor tendons is almost absent with extended PIP joints [30], the small load on the pulley outweighs the fact that the body weight is supported by only three fingers.

#### 4.3.3. Half-Crimp Grip

As expected, the grip position in the half-crimp, in general, lies between the crimp and the open-hand position, except for the MCP of the ring finger. However, there was still one individual participant who had a slightly higher PIP flexion angle measured in the half-crimp than in the crimp (CLIM_0001 left hand) and one individual who had slightly lower PIP flexion angles measured in the half-crimp than in the open-hand grip (CLIM_0005).

#### 4.3.4. Campusing

Campusing was both physically and technically the most demanding of all tasks, as no feet were used to support the body weight. This was expressed in the highest amount of joint movement during P2 and the highest intra-participant variability. One participant was unable to perform the campusing task. Overall, the self-selected grip position during campusing (crimp not allowed) was most similar to the half-crimp. One climber had a mean PIP angle of 29° during P2 when campusing, and all others laid within a range of 62°–88°. Regarding the individual climbers, most climbers (n = 7) spontaneously used a grip position that lies between their individual half-crimp and crimp position, whereas only three used a grip position that lies between their individual open-hand and half-crimp position.

### 4.4. Radial–Ulnar Deviation and Longitudinal Rotations of the Finger Joints

The finger PIP and DIP joints are considered hinge joints with one active degree of freedom (flexion–extension), and the MCP joints allow for flexion–extension and adduction–abduction movements. However, additional passive axial rotations (MCP, PIP, DIP) as well as passive lateral motions (PIP, DIP) seem to be important for hand function during grasping, as they increase pulp contact areas during pinching or facilitate adaptation of the hand surface to objects being held during larger grips [34,35,36]. Such passive finger joint rotations are not under direct voluntary control but rather occur due to the complex skeletal and ligamentous geometry [35,36] as well as external forces applied to the hand [34,35,37] and constraints through the shape of the object to be grasped [35]. When climbing, the acting forces are large compared to everyday gripping tasks and optimizing pulp contact areas seems to be particularly important in order to prevent slipping on small or difficult to grasp holds. However, the passive finger joint rotations during climbing were completely unknown because these small movements cannot be quantified with video cameras, and this is the first study to use motion capture on the fingers during climbing.

Regarding the position during the holding phase, we found mean radial deviations in the MCP of up to 20.4° and ulnar deviations of 20.0° in the PIP. Similarly, rotations along the longitudinal axis of the finger joints were in the range of −18.6° (MCP4, open-hand grip) to 21.6° (PIP4, campusing). Such high magnitudes of passive rotations may not be neglected, in particular, for a more in-depth understanding of the load on anatomical structures of the fingers during climbing movements.

Previous studies have examined the lateral stability of the fingers by applying a small lateral weight (40–250 g) [37,38]. They found passive ulnar deviation of 2.8° (range 1.2–9.9°) and radial deviation of 3.0° (0.5°–11.0°) in healthy index and middle finger PIP joints [37] or 7° in cadaver [38]. Passive lateral PIP deflections increased proportionally with applied load [37]. The PIP3 radial–ulnar deviation in our study was similar (−5.3° to 7.2°), whereas PIP4 lateral deviations of up to −20° (ulnar) were markedly larger in our study. Possible explanations are the higher acting forces during climbing; however, possible measurement artifacts also need to be taken into account, as discussed below.

The passive rotations were larger in the ring finger than in the middle finger, which is in agreement with previous studies that also found passive axial rotations of the MCP to be larger in the ring finger than in the middle finger (max. supination in MCP4 20°, in MCP3 14°) [35] and PIP passive pronation (6°) in the middle and supination (−9°) in the ring finger [39]. Unexpectedly, there was a kind of counter-movement between MCP4 and PIP4, with supination and radial deviation detected in the MCP4, while pronation and ulnar deviation were present in the PIP4 during all tasks. Possibly, the secondary rotations are influenced by how much the main rotation axis (flexion–extension) of the finger joints deviates from the orientation of the upper edge of the hand hold because this might cause a larger moment acting laterally or longitudinally on the finger joints. The influence of the orientation of the hand hold needs to be further examined in future studies.

Nevertheless, deflections appear to be relatively large, especially in the PIP joints. Effects of kinematic crosstalk or skin displacement artefacts need to be considered since secondary rotations are more susceptible to such errors; however, they cannot be quantified from the current data. For comparison, we also examined the lateral deflection and longitudinal rotations during the simple flexion–extension movements of the participating climbers. Averaged over all PIP and DIP joints, maximal radial deviation was 2.8° (4.9°), ulnar deviation 5.4° (5.1°), pronation 6.4° (4.1°) and supination 6.0° (4.1°). Thus, passive rotations in finger PIP and DIP joints during the climbing tasks were considerably larger, and therefore, it is suggested that these high passive rotations were, at least in part, caused by the climbing-specific loading and (grip-specific) kinematic constraints.

### 4.5. Limitations

#### 4.5.1. Climbing Moves

The climbing task itself might influence the joint angles. It was previously shown that altered foot position changed the maximum joint angles of the shoulder, hip, knee and ankle joints [8], but no comparable data are available for the joints of the hand. We used the same setup for all grip positions, which ensures comparability between the grip positions. However, based on our results, it is not possible to determine to what extent the climbing task and related moves influence the joint angles. The four performed climbing moves differed in length, leading to different body positions for each climbing move, which again possibly influenced grip positions during P2. The difficulty of the right-hand move was probably higher compared to the left due to a longer reach and a stationary hand on a smaller handhold. Further, the starting and the top holds were a lot bigger than the instrumented holds, allowing a more static grabbing (P1) of the right and a more static handhold release (P3) of the left hand. Uniform climbing moves, and handholds would have made the data for the right and the left hand more comparable. On the other hand, the variability of movements and handhold sizes represents a more realistic picture of sport climbing.

#### 4.5.2. Marker Visibility

As marker visibility was at a critical point and led to missing data, the number of participants for whom data could be extracted needs to be considered for data interpretation of each joint metric. In addition, measurement artefacts from skin displacement need to be taken into account, particularly for the interpretation of passive rotations.

## 5. Conclusions

It was possible to collect sufficient data, particularly for the holding phase, to gain new insights into finger kinematics during different climbing tasks. Better resolution of more recent optoelectronic motion capture systems will probably further improve data quality. As a result, optoelectronic motion capture proved to be practicable and reliable for motion analysis of the finger joints and wrists during sport climbing.

The joint angles obtained were consistent with the general descriptions of the common grip positions; however, the large inter-individual ranges of joint angles overlapped between the different grip positions, and some unexpected results were present in a few individuals despite all being experienced climbers. It seems that the different grip positions are adopted less distinctively during a real climbing task, in contrast to simulated/isolated gripping, but rather joint angles are influenced by various factors such as anthropometry and climbing tasks in addition to the requested grip positions. A variation of the movement tasks (distance, shape of the hold, foot position, inclination of the wall) in future studies will allow a more general consideration of the different grip positions.

Passive radial–ulnar positions, as well as rotations along the longitudinal axis of the finger up to ~20° were found. These high values indicate that these are relevant degrees of freedom of the finger joints in climbing and, therefore, also emphasize the importance of 3D recording of the joint angles.

Without precise instructions given, climbers might perform the open-hand grip with or without a little finger, leading to more or less PIP joint flexion in the middle and ring fingers. After pulley rupture, careful instruction not to use the little finger when restarting climbing is important, as this poses less stress on the convalescent pulley.

## Figures and Tables

**Table 1:** Anthropometric data and redpoint (RP) climbing level of the participants according to IRCRA grading [19]. Plot color refers to the color used for kinematic plots of individual climbers; hence, the climbers can be identified by this color in Figure A4, Figure A5 and Figure A6. Participant “CLIM_0011” had to be excluded due to technical problems affecting kinetic data acquisition.

Participant	Sex	Age	Weight	Height	Climbing Experience	Training Hours Per Week	Max RP Bouldern IRCRA	Max RP Lead Climbing IRCRA	Plot Color
		[years]	[kg]	[cm]	[years]	[h]	[max]	[max]	
CLIM_0001	F	25	69	168	17	15	26.5	23	
CLIM_0002	M	28	72	185	5	12	23.5	n.a.	
CLIM_0003	M	31	67	174	10	6	26.5	23	
CLIM_0004	M	32	66	174	12	12	28.5	29	
CLIM_0005	M	38	75	174	15	6	23.5	21	
CLIM_0006	M	45	65	179	27	6	27.5	27	
CLIM_0007	M	51	70	180	30	3.5	26.5	27	
CLIM_0008	M	30	74	185	15	3	24.5	25	
CLIM_0009	M	20	70	176	10	14	26.5	23	
CLIM_0010	M	23	69	179	6	10	25.5	18	
CLIM_0012	M	36	73	187	20	8	27.5	25	
Average	1F 10M	32.6	70.0	178.3	15.2	8.7	26.0	24.1	
SD		9.1	3.0	5.5	7.6	4.0	1.6	3.0	

**Table 2:** Flexion(+)–extension(-) ROM [°] (SD) during the grabbing (P_1_), holding (P_2_) and releasing (P_3_) phase averaged over all participants and conditions. Øn reflects the average number of participants with valid trials for ROM calculation; however, n might differ between conditions. It was specified with a footnote only if Øn < 11.

	Wrist	MCP3	MCP4	PIP3	PIP4	DIP3	DIP4	Overall Mean
P_1_	14.2 (2.4)	16.1 (2.3)	16.8 (2.2) ^1^	20.5 (7.0) ^1^	25.3 (7.6) ^1^	19.2 (4.8) ^1^	18.1 (6.9)	18.6
P_2_	12.7 (3.1)	9.2 (2.0)	8.5 (1.2) ^1^	7.9 (2.6) ^1^	10.0 (2.8) ^1^	9.3 (3.0) ^2^	9.1 (2.4) ^1^	9.5
P_3_	28.1 (2.5)	22.3 (6.8)	26.5 (6.0) ^1^	6.7 (1.0) ^1^	7.8 (3.3) ^2^	8.1 (3.9) ^3^	7.1 (4.5) ^2^	15.2

^1^ Øn = 10; ^2^ Øn = 9; ^3^ Øn = 8.

**Table 3:** Radial(+)–ulnar(-) deviation ROM [°] (SD) during the grabbing (P_1_), holding (P_2_) and releasing (P_3_) phase averaged over all participants and conditions. Øn reflects the average number of participants with valid trials for ROM calculation; however, n might differ between conditions. It was specified with a footnote only if Øn < 11.

	Wrist	MCP3	MCP4	PIP3	PIP4	DIP3	DIP4	Overall Mean
P_1_	8.5 (2.3)	8.5 (1.6)	7.9 (1.3) ^1^	10.4 (1.9) ^1^	12.2 (2.5) ^1^	6.0 (2.0) ^1^	5.5 (1.3)	8.4
P_2_	11.5 (1.9)	5.4 (1.5)	4.8 (1.3) ^1^	8.0 (2.3) ^1^	8.4 (2.0) ^1^	5.1 (2.6) ^2^	5.1 (1.7) ^1^	6.9
P_3_	13.2 (2.4)	4.9 (0.6)	5.0 (1.3) ^1^	4.8 (0.6) ^1^	5.2 (1.3) ^2^	2.9 (1.2) ^3^	2.7 (1.1) ^2^	5.5

^1^ Øn = 10; ^2^ Øn = 9; ^3^ Øn = 8.

**Table 4:** Pronation(+)–supination(-) ROM [°] (SD) during the grabbing (P_1_), holding (P_2_) and releasing (P_3_) phase averaged over all participants and conditions. Øn reflects the average number of participants with valid trials for ROM calculation; however, n might differ between conditions. It was specified with a footnote only if Øn < 11.

	Wrist	MCP3	MCP4	PIP3	PIP4	DIP3	DIP4	Overall Mean
P_1_	7.2 (1.9)	10.1 (2.1)	12.8 (2.9) ^1^	11.5 (1.9) ^1^	13.4 (2.8) ^1^	10.3 (2.3) ^1^	10.6 (1.9)	10.8
P_2_	10.0 (3.6)	7.3 (2.5)	8.5 (2.0) ^1^	9.1 (2.6) ^1^	9.5 (2.4) ^1^	9.0 (3.1) ^2^	8.9 (2.2) ^1^	8.9
P_3_	6.8 (1.3)	9.7 (1.9)	8.2 (1.6) ^1^	5.8 (1.2) ^1^	6.7 (2.2) ^2^	5.1 (1.4) ^3^	4.6 (1.5) ^2^	6.7

^1^ Øn = 10; ^2^ Øn = 9; ^3^ Øn = 8.

**Table 5:** Mean (SD) flexion(+)–extension(-) joint angles [°] during the holding phase (P2) averaged over all climbers. N refers to the number of participants with at least one available value for the corresponding joint parameter. Superscripted letters refer to statistically significant differences between the left and right hand (A) as well as between corresponding joints of the middle and ring finger within the same hand (a–e).

Grip Type	Side	Wrist			MCP3			MCP4			PIP3			PIP4			DIP3			DIP4		
Crimp	left	−36.6	(7.1)	n = 11 ^A^	43.9	(9.1)	n = 11 ^a^	30.2	(10.9)	n = 10 ^a^	84.6	(9.9)	n = 9	87.4	(14.8)	n = 10	−23.7	(7.3)	n = 9	−16.7	(10.8)	n = 11
	right	−27.6	(5.8)	n = 11 ^A^	36.8	(8.3)	n = 11 ^b^	23.2	(10.2)	n = 11 ^b^	89.0	(12.0)	n = 11	85.6	(14.4)	n = 7	−28.3	(9.8)	n = 6	−11.2	(16.4)	n = 4
Half-crimp	left	−17.4	(6.9)	n = 11	12.8	(10.4)	n = 11	8.7	(14.6)	n = 10	75.0	(13.3)	n = 9	68.3	(15.2)	n = 10	−13.0	(7.2)	n = 9	−5.8	(6.0)	n = 11
	right	−14.4	(7.7)	n = 11	14.9	(11.5)	n = 11	9.9	(12.2)	n = 11	69.6	(14.6)	n = 10	66.2	(13.1)	n = 10	−10.6	(7.9)	n = 10	−7.3	(9.8)	n = 10
Open-hand	left	−10.2	(9.2)	n = 11	4.0	(8.2)	n = 11	10.5	(11.6)	n = 10	58.6	(13.2)	n = 10	31.7	(29.0)	n = 10	−2.5	(10.4)	n = 10 ^d^	16.5	(18.8)	n = 11 ^d^
	right	−4.4	(7.6)	n = 11	4.3	(11.9)	n = 11	12.7	(13.6)	n = 11	49.4	(24.7)	n = 11 ^c^	17.9	(24.0)	n = 11 ^c^	3.8	(16.1)	n = 11 ^e^	19.4	(14.9)	n = 11 ^e^
Campusing	left	−18.0	(7.8)	n = 10	14.8	(13.1)	n = 10	8.2	(14.5)	n = 9	72.5	(15.6)	n = 9	66.2	(19.9)	n = 9	−7.9	(9.0)	n = 9	−0.6	(9.2)	n = 10
	right	−20.0	(6.5)	n = 10	18.0	(12.9)	n = 10	9.4	(13.1)	n = 10	70.7	(17.2)	n = 9	68.6	(19.1)	n = 10	−7.3	(8.8)	n = 8	0.5	(9.0)	n = 9

^A:^*p* = 0.0037, ^a:^ *p* = 0.009, ^b:^ *p* = 0.0027, ^c:^ *p* = 0.026, ^d:^ *p* = 0.011, ^e:^ *p* = 0.029.

**Table 6:** Minimum and maximum within-participant average of the flexion(+)–extension(-) joint angles of the ring and middle finger PIPs during the holding phase.

Grip Type		lPIP3	lPIP4	rPIP3	rPIP4
Crimp	Min	67.4	58.3	61.9	58.8
	Max	96.4	106.2	105.3	101.4
Half-crimp	Min	43.5	27.9	41.2	38.2
	Max	85.7	84.2	86.9	78.2
Open-hand	Min	39.9	0.8	−4.3	−2.9
	Max	79.9	78.5	82.0	69.1
Campusing	Min	37.6	20.0	34.5	23.6
	Max	87.7	87.8	88.8	91.4

## Data Availability

The datasets presented in this article are not readily available because the data are part of an ongoing study.

## Appendix A

**Table A1:** Mean (SD) radial–ulnar deviation joint angles [°] during the holding phase averaged over all climbers (n identical to Table 5). Superscripted numbers refer to statistically significant differences between the middle and ring fingers.

Grip Type	Side	Wrist		MCP3		MCP4		PIP3		PIP4		DIP3		DIP4	
Crimp	left	−10.2	(8.8)	13.3	(11.1)	18.1	(13.2)	−1.5	(6.3) ^1^	−12.9	(6.3) ^1^	−1.6	(3.9)	−0.2	(7.4)
	right	−12.0	(6.8)	12.4	(9.9) ^3^	20.4	(6.1) ^3^	−0.3	(9.3) ^2^	−13.8	(10.6) ^2^	5.6	(10.3)	0.2	(13.4)
Half-crimp	left	−4.7	(7.4)	4.7	(13.3)	12.7	(13.2)	1.0	(11.3) ^4^	−17.3	(8.5) ^4^	−2.0	(3.4)	−0.6	(6.3)
	right	−7.2	(8.6)	1.3	(13.5) ^6^	13.6	(9.7) ^6^	7.2	(10.3) ^5^	−18.8	(10.9) ^5^	1.2	(5.2)	−1.5	(7.5)
Open-hand	left	−11.6	(7.1)	9.6	(12.3)	15.9	(11.8)	−4.3	(11.5)	−14.9	(12.6)	−0.9	(5.3)	0.2	(6.5)
	right	−14.2	(8.1)	6.4	(11.3)	14.7	(7.4)	−1.5	(6.7)	−4.7	(11.0)	1.5	(4.5)	0.0	(5.3)
Campusing	left	−7.0	(8.0)	11.8	(10.7)	15.8	(12.3)	−5.3	(7.6) ^7^	−18.6	(8.6) ^7^	−2.1	(5.0)	0.8	(8.2)
	right	−3.7	(10.1)	8.4	(13.0)	18.5	(8.6)	3.3	(10.9) ^8^	−20.0	(10.0) ^8^	−2.7	(3.7)	−3.1	(7.4)

^1^ *p* = 0.007; ^2^ *p* = 0.014; ^3^ *p* = 0.033; ^4^ *p* = 0.006; ^5^ *p* < 0.001; ^6^ *p* = 0.025; ^7^ *p* = 0.011; ^8^ *p* < 0.001.

**Table A2:** Mean (SD) pronation–supination joint angles [°] during the holding phase averaged over all climbers (n identical to Table 5). Superscripted numbers refer to statistically significant differences between the middle and ring fingers.

Grip Type	Side	Wrist		MCP3		MCP4		PIP3		PIP4		DIP3		DIP4	
Crimp	left	0.8	(4.0)	−1.4	(4.5) ^9^	−9.8	(6.2)^9^	1.2	(6.4)^1^	12.6	(6.3)^1^	7.1	(5.7)	0.6	(7.0)
	right	3.7	(5.4)	0.7	(5.9) ^10^	−9.6	(6.0)^10^	0.5	(8.4)^2^	14.1	(10.6)^2^	12.6	(13.4)	4.8	(6.1)
Half-crimp	left	3.7	(4.6)	6.0	(4.1) ^11^	−12.0	(7.4)^11^	−1.1	(12.6)^3^	15.3	(8.2)^3^	4.7	(10.7)	−1.9	(7.8)
	right	5.3	(6.4)	8.9	(6.2) ^12^	−13.4	(6.7)^12^	−6.4	(11.1)^4^	19.8	(10.4) ^4^	3.2	(5.0)	5.0	(7.3)
Open-hand	left	3.5	(3.4)	0.1	(4.2) ^13^	−17.3	(7.0)^13^	3.2	(10.5)^5^	20.2	(7.4)^5^	4.7	(5.7)	−0.1	(6.6)
	right	7.2	(5.6)	−1.4	(8.1) ^14^	−18.6	(5.8)^14^	5.0	(7.6)^6^	17.4	(6.3)^6^	3.0	(4.8)	5.9	(3.4)
Campusing	left	8.2	(4.0)	6.5	(5.7) ^15^	−8.5	(5.0)^15^	4.9	(7.3)^7^	17.6	(7.7)^7^	3.6	(7.1)	−0.5	(5.7)
	right	7.8	(4.9)	9.1	(9.4) ^16^	−8.1	(7.5)^16^	−1.8	(11.1)^8^	21.6	(8.9)^8^	3.9	(6.1)	4.9	(3.9)

^1^ *p* = 0.006; ^2^ *p* = 0.011; ^3^ *p* = 0.018; ^4−6^ *p* < 0.001; ^7^ *p* = 0.009; ^8^ *p* < 0.001; ^9^ *p* = 0.002; ^10−16^ *p* < 0.001.
